# The LIM-Only Protein FHL2 Mediates Ras-Induced Transformation through Cyclin D1 and p53 Pathways

**DOI:** 10.1371/journal.pone.0003761

**Published:** 2008-11-19

**Authors:** Charlotte Labalette, Yann Nouët, Florence Levillayer, Carolina Armengol, Claire-Angélique Renard, Guillaume Soubigou, Tian Xia, Marie-Annick Buendia, Yu Wei

**Affiliations:** 1 Institut Pasteur, Unité d'Oncogenèse et Virologie Moléculaire, Paris, France; 2 Inserm U579, Paris, France; 3 Institut Pasteur, PT “Puce à AND”, Paris, France; MGH/Harvard University, United States of America

## Abstract

**Background:**

Four and a half LIM-only protein 2 (FHL2) has been implicated in multiple signaling pathways that regulate cell growth and tissue homeostasis. We reported previously that FHL2 regulates cyclin D1 expression and that immortalized *FHL2*-null mouse embryo fibroblasts (MEFs) display reduced levels of cyclin D1 and low proliferative activity.

**Methodology/Principal Findings:**

Here we address the contribution of FHL2 in cell transformation by investigating the effects of oncogenic Ras in *FHL2*-null context. We show that H-RasV12 provokes cell cycle arrest accompanied by accumulation of p53 and p16^INK4a^ in immortalized *FHL2^−/−^* MEFs. These features contrast sharply with Ras transforming activity in wild type cell lines. We further show that establishment of *FHL2*-null cell lines differs from conventional immortalization scheme by retaining functional p19^ARF^/p53 checkpoint that is required for cell cycle arrest imposed by Ras. However, after serial passages of Ras-expressing *FHL2^−/−^* cells, dramatic increase in the levels of D-type cyclins and Rb phosphorylation correlates with the onset of cell proliferation and transformation without disrupting the p19^ARF^/p53 pathway. Interestingly, primary *FHL2*-null cells overexpressing cyclin D1 undergo a classical immortalization process leading to loss of the p19^ARF^/p53 checkpoint and susceptibility to Ras transformation.

**Conclusions/Significance:**

Our findings uncover a novel aspect of cellular responses to mitogenic stimulation and illustrate a critical role of FHL2 in the signalling network that implicates Ras, cyclin D1 and p53.

## Introduction

Four and a half LIM-only protein 2 (FHL2) is a multi-functional protein that shuttles between focal adhesion sites and nucleus. Focal adhesions are specialized adhesive junctions where integrin receptors for extracellular matrix (ECM) are concentrated [1]. FHL2 interacts with α- and β-integrin subunits and focal adhesion kinase (FAK) [2], [3] and is involved in assembly of ECM proteins on the cell surface [4]. Deregulation of genes involved in focal adhesion, ECM-receptor interaction and response to external stimuli has been evidenced in mouse embryo fibroblasts (MEF) deficient for FHL2 [5], suggesting that FHL2 plays important roles in tissue physiology and homeostasis and in the transduction of signals emitted from extracellular environment. Indeed, in response to RhoA signals or serum stimulation, FHL2 is translocated to the nucleus [6], [7] where it acts as either transcription coactivator or corepressor in interaction with numerous transcription factors including the androgen receptor, AP1, CREB, PLZF, SKI, β-catenin, FOXO1, Runx2 and serum response factor (SRF) [7], [8], [9], [10], [11], [12], [13], [14], [15], [16]. The ability of FHL2 to assemble multiple partners into protein complexes is attributed to its LIM-domain structure that serves as protein binding interface through double zinc finger motifs [17]. Thus, FHL2 participates in regulating expression of a large spectrum of genes involved in cell proliferation, differentiation, transformation and apoptosis. Despite absence of gross defects during development, *FHL2*-deficient mice harbor cardiac hypertrophy in response to β-adrenergic stimulation, osteopenia due to decreased activity of osteoblasts and wound healing defect [15], [18], [19].

Several lines of evidence have implicated FHL2 in the control of cell proliferation. We have recently shown that FHL2 physically occupies the cyclin D1 promoter and directly stimulates cyclin D1 transcription [5]. Accordingly, we found reduced expression levels of cyclin D1 in MEFs isolated from *FHL2*-null mice. After spontaneous immortalization, these cells exhibited weak proliferative capacity associated with low expression levels of all D-type cyclins and cyclin E, as well as cyclin-dependent kinase (Cdk) inhibitors, in particular p16^INK4a^ [5], [20]. It has been shown that FHL2 can inhibit the antiproliferative functions of the transcription factor E4F1 by inhibiting the transcription repressor function and p53 binding activity of E4F1 [21]. In gastric and colon cancer cell lines, suppression of FHL2 inhibited anchorage-dependent and -independent cell growth, and tumor formation in nude mice xenograft [22]. In line with its effects on cell growth, overexpression of FHL2 has been detected in various types of cancer [13], [22], [23], [24]. Moreover, androgen-induced nuclear accumulation of FHL2 in highly malignant prostate carcinoma has been linked to disease progression and poor outcome [6], [25].

In this study, we sought to gain further insight into the role of FHL2 in neoplastic transformation by investigating the effects of oncogenic Ras in a *FHL2*-deficient context. In primary rodent cells, activated Ras mutant induces premature senescence associated with activation of p16^INK4a^ and p53 [26]. Cellular senescence involves an essentially irreversible cell cycle arrest that is associated with heterochromatin formation mediated by the retinoblastoma (Rb) tumor suppressor [27]. The ARF/p53 and p16^INK4a^/Rb pathways are clearly critical for establishing the senescence growth arrest, and primary MEFs deficient for p19^ARF^ and p53 exhibit resistance to Ras-induced senescence and are susceptible to transformation by activated Ras alone [28]. In general, cooperation of Ras and additional “immortalizing” factors is required for transformation of primary rodent cells. However, oncogenic Ras alone readily transforms immortalized cells lines, since 80% of these cells contain mutant p53, whereas most others sustain biallelic loss of the *INK4a/ARF* locus [29].

Here, we report that H-RasV12 provokes a cell cycle arrest in spontaneously immortalized *FHL2*-null cells, in contrast to cell transformation in wild type (wt) counterparts. The cell growth arrest induced by Ras is controlled by p53 which remains wt after spontaneous immortalization of *FHL2*-null cells. We found that under continuous Ras-mediated mitogenic stimulation, D-type cyclins are dramatically increased in *FHL2*-deficient cells, resulting in reversal of the growth arrest without disrupting the ARF/p53 checkpoint.

## Results

### Absence of foci formation in immortalized *FHL2^−/−^* fibroblasts upon H-RasV12 expression

**Figure 1:**
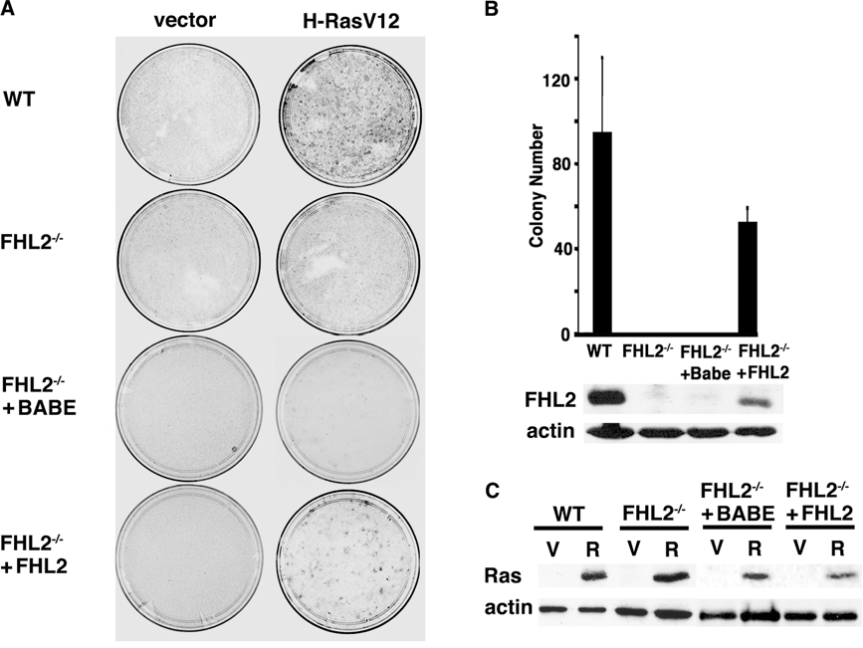
Absence of foci formation in immortalized *FHL2^−/−^* MEFs upon expression of H-RasV12. (A) Focus formation assay of MEFs transiently transfected with H-RasV12 or empty vector. Plates were stained with Giemsa at 3 weeks after transfection. Data are representative of at least 3 independent experiments using 3 spontaneously immortalized clones. To restore FHL2 expression, primary *FHL2^−/−^* MEFs were infected with a retrovirus expressing FHL2 (pBabeFHL2puro) and cultured according to the 3T3 protocol. Two *FHL2*-restored *FHL2^−/−^* cell pools were obtained and used in the study. pBabe vector was used as a control (Babe). (B) Chart depicting at least three independent experiments. Mean colony numbers±standard deviation are shown. Bottom: Immunoblot analysis of FHL2 expression in *FHL2*-restored cell line. (C) Analysis of H-RasV12 expression in MEFs transfected with either empty (V) or H-RasV12 (R) expression vector. Samples were processed for immunoblotting 48 h after transfection. Actin was used as loading control.

We have recently reported the establishment of spontaneously immortalized cell lines from primary MEFs derived from *FHL2^−/−^* embryos [30] by using a 3T3 protocol [5]. To assess the effects of FHL2 deficiency on cell transformation, we expressed the oncogenic form H-RasV12 in three independent immortalized wt and *FHL2^−/−^* MEF cell lines. Transiently transfected cells were analyzed for the loss of contact inhibition by focus formation assays. As expected, numerous colonies were detected in wt fibroblasts, but strikingly, no colonies developped in immortalized *FHL2^−/−^* MEFs (Figs. 1A and 1B), despite similar levels of Ras expression in these cell lines as shown by immunoblotting (Fig. 1C). To confirm that FHL2 deficiency was directly involved in this phenotype, we then re-expressed FHL2 from a retroviral vector in primary *FHL2^−/−^* MEFs at early passage. Continuous passage of *FHL2*-restored MEFs readily gave rise to established 3T3 cell lines in which the levels of FHL2 protein were significantly lower than in wt MEFs (Fig. 1B). Transfection of the Ras vector in *FHL2*-restored MEF lines resulted in colony formation, although transformation efficiency was about half of that in wt MEFs (Figs. 1A and 1B). Thus, low level FHL2 expression was sufficient to restore the capacity of immortalized MEFs to undergo deregulated growth and loss of contact inhibition in response to oncogenic Ras. These results indicate that FHL2 deficiency impairs Ras-induced transformation of immortalized MEFs.

### H-RasV12 induces a cell cycle arrest in immortalized *FHL2^−/−^* MEFs

**Figure 2:**
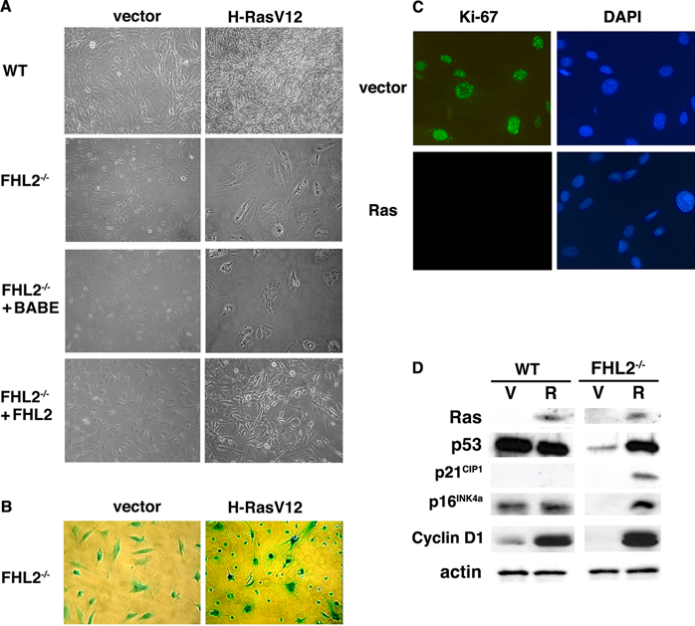
H-RasV12 induces cell cycle arrest in immortalized *FHL2^−/−^* MEFs. (A) Immortalized wt, *FHL2^−/−^* and *FHL2^−/−^-*restored MEFs were infected with pBabe-RasV12 or control pBabe retrovirus and selected for drug resistance. (B) All *FHL2^−/−^* cells were positive in β-galactosidase assay due to the insertion of *Lac Z* cDNA at the *FHL2* locus. (C) Ki-67 immunofluorescence analysis in vector- and H-RasV12-transduced *FHL2^−/−^* MEFs. Nuclei were counterstained with DAPI. (D) Expression of Ras, p53, p21^CIP1^, p16^INK4a^ and cyclin D1 in H-RasV12- (R) or vector- (V) transduced wt and *FHL2^−/−^* cell lines. Equal amounts of protein extracts were analyzed by immunoblotting.

It has been shown that oncogenic Ras can induce either transformation, senescence or apoptosis, depending on the cell context [31]. To further investigate the effects of Ras in the context of FHL2 deficiency, immortalized wt, *FHL2^−/−^* and *FHL2-*restored cells were infected with pBabe-RasV12 or control pBabe retrovirus and selected for drug resistance. Both Ras-transduced wt and *FHL2-*restored MEFs exhibited thin, elongated, and spindle-like morphology and loss of contact inhibition characteristic of transformed fibroblasts (Fig. 2A). Moreover, when injected s.c. into BALB/c nude mice, Ras-transduced wt and *FHL2*-restored fibroblasts generated large tumors in 6/6 mice. In control experiment, none of the 6 mice injected with vector-transduced wt or *FHL2-*restored cells developed tumors.

In sharp contrast, H-RasV12 transduction produced very different morphological changes in immortalized *FHL2^−/−^* cell lines. Cells in these cultures were large, flat and nonrefractile (Fig. 2A), evoking a senescent-like morphology that could not be detected in transient assays, in which non-transfected cells were predominant (see Fig. 1A). Three independent *FHL2^−/−^* lines displayed similar morphology upon Ras expression (data not shown). However, β-galactosidase activity [32] could not be tested as a senescence-associated marker in this system, because the *lacZ* cDNA was inserted at the *FHL2* locus for generating *FHL2*-null mice [30], resulting in β-galactosidase activity in all *FHL2^−/−^* cells even at acidic conditions (Fig. 2B).

To better characterize the phenotype of H-RasV12-transduced immortalized *FHL2^−/−^* cells, we first examined whether these cells were growth arrested by monitoring their proliferative activity, using immunostaining with a Ki-67 specific antibody. While positive signals were observed in *FHL2^−/−^* cells transduced with the pBabe vector, no Ki-67-positive cell was detected in H-RasV12-transduced *FHL2^−/−^* cultures (Fig. 2C). We then assessed the expression of genes that are specifically associated with the G1 arrest induced by oncogenic Ras in fibroblasts, namely p53 and the cyclin–dependent kinase (Cdk) inhibitors p21^CIP1^ and p16^INK4a^ that promote cell cycle arrest via the Rb tumor suppressor pathway. Immunoblotting analysis revealed that expression of oncogenic Ras was associated with increased levels of p53, p21^CIP1^ and p16^INK4a^ in *FHL2^−/−^* MEFs, but not in wt cells (Fig. 2D). The basal level of p16^INK4a^ was extremely low in *FHL2^−/−^* cells [5] (Fig. 2D). It has been demonstrated that high levels of p16^INK4a^ provide a dominant second barrier to the unlimited growth of human fibroblasts [33]. The induction of p16^INK4a^ by Ras in *FHL2^−/−^* cells may provide an alternative mechanism of p53 activation for cell cycle exit. In addition, consistent with previous reports showing that cyclin D1 is activated by oncogenic Ras in both senescent and transformed fibroblasts [26], [34], we found equally high level expression of cyclin D1 in wt and *FHL2^−/−^* cells (Fig. 2D). The fold increase of cyclin D1 by Ras was more dramatic in *FHL2^−/−^* cells, given its low basal level (Fig. 2D) [5].

Based on the observed morphological and biochemical changes, it seems likely that Ras-induced cell cycle arrest of immortalized *FHL2^−/−^* cells was triggered by a senescent-like program that involves p53 and p16^INK4a^. This cell cycle arrest corresponds to the model of hypermitogenic arrest that is associated with a ‘large cell’ morphology and protein synthesis [35].

### Immortalized *FHL2^−/−^* MEFs retain wt *p53* and an intact *INK4a/ARF* locus

**Figure 3:**
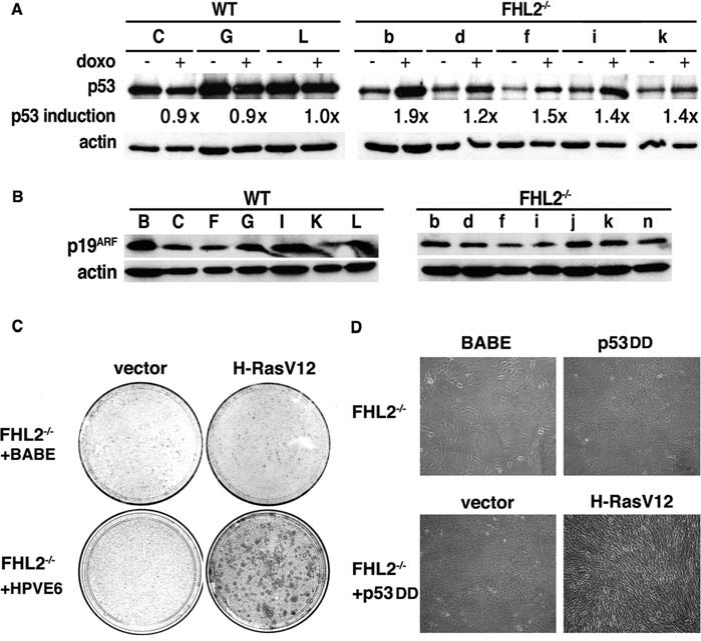
Analysis of the p53/ARF pathway in immortalized *FHL2^−/−^* MEFs. (A) Functional analysis of the p53 protein. After induction by doxorubicin (doxo), p53 was immunoprecipitated from immortalized MEFs and analyzed by immunoblotting. Wild type clones are indicated by capital letters and *FHL2^−/−^* clones by lower-case letters. p53 induction was determined as the ratio of the relative p53/actin intensities after doxo treatment to p53/actin intensities without doxo in each cell line. (B) Western blot analysis of p19^ARF^ expression in seven immortalized clones of each FHL2 genotype. (C) Human papillomavirus E6 oncoprotein restores Ras-mediated transformation in immortalized *FHL2^−/−^* MEFs. Cells were infected with E6-expressing retrovirus (HPVE6) or control virus (Babe), and then transiently transfected with H-RasV12 plasmid or control vector, cultured for 3 weeks without splitting and stained. Data are representative of three independent experiments in two independent E6-transduced *FHL2^−/−^* MEF clones. (D) Dominant negative p53 (p53DD) was expressed alone or coexpressed with H-RasV12 by retroviral vectors in immortalized *FHL2^−/−^* MEFs.

Previous studies have shown that 80% of spontaneously immortalized MEF cell lines harbor mutant p53 alleles, whereas most of the others sustain biallelic loss of the *INK4a/ARF* locus [29]. However, the low basal level of p53 and its activation by H-RasV12 in immortalized *FHL2^−/−^* MEFs suggest that these cells may harbor wt p53 (Fig. 2D). We assessed the p53 status of immortalized MEFs by treating cells with doxorubicin (doxo), a DNA damage-inducing drug that induces the up-regulation of wt p53, but has no effect on the already stabilized p53 mutants. Detection of p53 was optimized by immunoprecipitating the protein from cell lysates before immunoblotting. In established wt cell lines, basal p53 levels were high in the absence of any stimulus, and did not increase in response to doxo (Fig. 3A), reflecting the hallmarks of p53 mutants. In sharp contrast, p53 was produced at low levels and was significantly induced by doxo in all five *FHL2^−/−^* cell lines (Fig. 3A), consistent with the presence of wt p53. We therefore went on to analyze the p53 gene by direct PCR sequencing of genomic DNA in four independent *FHL2^−/−^* cell lines. Using a set of five PCR fragments covering exons 4 to 10, both strands of each exon were sequenced. Of four *FHL2^−/−^* cell lines analyzed, we did not detect any mutation, which confirms the wt status of p53 in immortalized *FHL2^−/−^* cells.

Analyses of p19^ARF^ expression in immortalized *FHL2*-null cell lines reproducibly showed the presence of the protein in all cell lines, regardless of FHL2 genotype (Fig. 3B). However, the p19^ARF^ protein was generally present in larger amounts in wt than in *FHL2^−/−^* cell lines, in agreement with previous reports showing that p19^ARF^ is up-regulated in cells with mutant p53 [36]. As reported previously, low levels of p16^INK4a^ were detected in all clones [5]. Based on these observations, we conclude that the *INK4a/ARF* locus remained intact in these cells. Disruption of p19^ARF^ or p53 is therefore not required for immortalization of FHL2^−/−^ MEFs.

### Microarray analysis shows up-regulation of p53 target genes in *FHL2*-deficient cells

**Figure 4:**
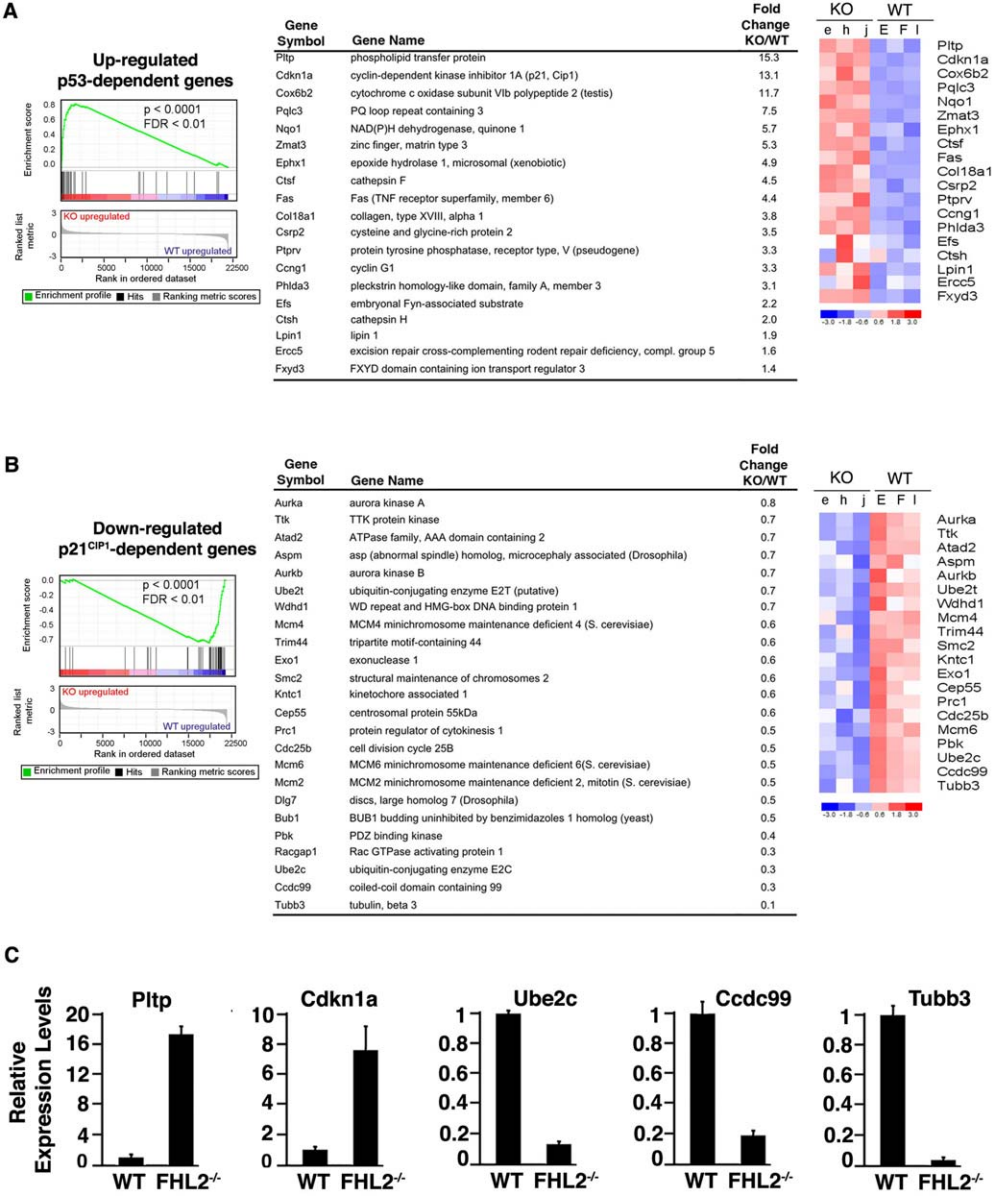
Expression of p53 and p21^CIP1^ target genes in *FHL2*-null cells (A) Left: GSEA plot using a gene set of up-regulated p53-dependent genes from the Molecular Signatures Database (MSigDB) (NES = 2.35, FDR<0.01, *p*<0.0001). Enrichment score (ES) reflects the correlation of the gene set with FHL2^−/−^ (KO) and WT genotypes. Middle: Core group of genes of the up-regulated p53-dependent gene set that contributed to the ES. The fold change (KO/WT) value for genes that have more than one probe set corresponds to the mean value. Right: Expression profile of the deregulated genes at fold change ≥1.4 in 3 different clones for each genotype. Data are plotted as a heatmap where red and blue correspond to high and low expression in log_2_-transformed scale. (B) GSEA Analysis using down-regulated p21^CIP1^-dependent gene set of MSigDB (NES = −2.25, FDR<0.01, *p*<0.0001). Expression profile of the top down-regulated genes at fold change ≤0.8 that are differently expressed in the FHL2^−/−^ compared to wt clones. Abbreviations: NES: normalized enrichment score, FDR: false discovery rate. (C) Quantitative RT-PCR analysis of Cdkn1a (p21^CIP1^), Pltp, Ube2c, Ccdc99, Tubb3 transcripts in three independent wt and *FHL2^−/−^* immortalized clones. Data were normalized to 18S RNA. Results are expressed as relative expression of each gene/18S RNA assuming wt cells as 1.0. Average values and standard deviations for three independent experiments are shown.

To confirm that the p53 pathway remained functional in *FHL2^−/−^* cell lines, we assessed the expression of p53 target genes at the whole-genome level by comparing gene expression profiles of three *FHL2^−/−^* cell lines with those of three immortalized wt MEF clones using Affymetrix Mouse Genome 430 2.0 microarray[5]. Up-regulation of p53 target genes in *FHL2^−/−^* cells was evidenced by Gene Set Enrichment Analysis (GSEA), a computational method for assessing enrichment of a predefined gene list in one class compared with another [37]. Using a list of thirty one p53 target genes of the Molecular Signatures Database (MSigDB) [38] as gene set, we found that 61% of these genes were significantly up-regulated in *FHL2^−/−^* cells (Fig. 4A). Specifically, several well-studied p53 target genes such as p21^CIP1^, Fas and cyclin G1 were increased in cells lacking FHL2. As the Cdk inhibitor p21^CIP1^ is a downstream effector of p53, we then analyzed the expression of genes that are targeted by p21^CIP1^ in *FHL2^−/−^* cells. For GSEA, we used a set of 39 genes that were down-regulated after ectopic expression of p21^CIP1^ in ovarian cancer cell lines [39], and found decreased expression in 61% of these genes in *FHL2^−/−^* MEFs (Fig. 4B). It included genes encoding aurora kinases, Mcm proteins, Kntc1, Cep55 and Cdc25b that are implicated in DNA replication, activation of M-phase, kinetochore assembly and chromosome segregation. Downregulation of these genes is consistent with the known effects of p21^CIP1^ on G1 and G2/M phase arrest [40]. Real-time RT-PCR analysis confirmed altered expression of p53 targets such as p21^CIP1^ and Pltp, and down-regulation of p21^CIP1^ downstream genes such as Ube2c, Ccdc99 and Tubb3 in *FHL2^−/−^* cells (Fig. 4C). Taken together, genome-wide analysis firmed up the functional activity of p53 and provided insight into the impact of functional p53 on gene expression in immortalized *FHL2*-null cells.

### Inactivation of p53 confers susceptibility to Ras-induced transformation in *FHL2^−/−^* cells

Integrity of the p19^ARF^/p53 pathway is required for induction of cellular senescence by oncogenic Ras [41]. To determine whether p53 inactivation could alter the effects of H-RasV12 expression in *FHL2^−/−^* MEFs, we first used the E6 oncoprotein of human papillomavirus-16 that causes p53 degradation and abolishes p53-mediated checkpoints. Retroviral transduction of E6 before transient transfection of immortalized *FHL2^−/−^* cell lines with H-rasV12 resulted in efficient colony formation (see Fig. 3C), thus allowing efficient transformation of these cells. Moreover, co-expression of a dominant negative form of p53, p53DD [42] and H-RasV12 by retroviral vectors in *FHL2^−/−^* cells resulted in thin, elongated, and spindle-like transformed morphology (see Fig. 3D). When injected s.c. into BALB/c nude mice, Ras- and p53DD-cotransduced *FHL2-null* fibroblasts induced tumor formation in 4/4 mice, whereas no tumor developped in 4 mice injected with *FHL2^−/−^* cells transduced with p53DD alone. Thus, p53 inactivation was sufficient to restore the susceptibility of immortalized *FHL2^−/−^* MEFs to Ras-mediated transformation.

### The cell cycle arrest induced by H-RasV12 in immortalized *FHL2^−/−^* MEFs is reversible

**Figure 5:**
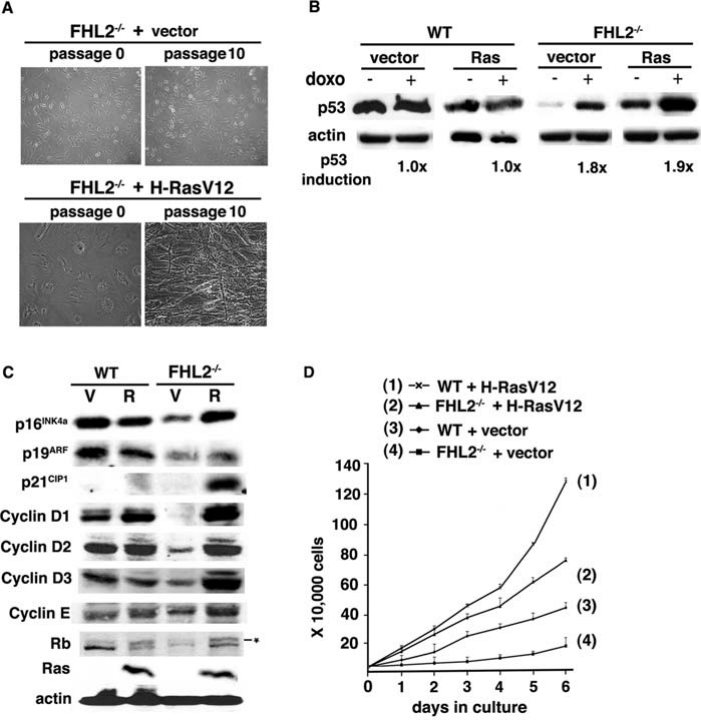
Reversal of the cell cycle arrest in H-RasV12-transduced *FHL2^−/−^* MEFs. (A) The indicated cell lines transduced by H-RasV12 or control vector are shown at cell cycle arrest stage (passage 0) and after reversal of the cell cycle arrest (passage 10). (B) Analysis of the p53 protein in cells at passage 10 after H-RasV12 transduction. Cells were treated with doxorubicine as described in Fig. 3A. (C) Western blot analysis of cell cycle regulators in wt and *FHL2^−/−^* cell lines at passage 10 after transduction with H-RasV12 (R) or empty vector (V). (D) Growth curves of the indicated cell lines after reversal. Equal numbers of cells were initially plated in triplicate wells, and cells were counted at indicated times. Mean values±standard deviation from three independent experiments are shown. Data presented are representative of 3 independent H-RasV12- and vector-expressing MEF lines.

The morphology of Ras-transduced *FHL2^−/−^* cells evoked cellular senescence that involves an essentially irreversible cell cycle arrest. When cultured with the 3T3 protocol, however, Ras-transduced *FHL2^−/−^* cells resumed proliferation after 5 passages. At passage 10, thin and spindle-like morphology characteristic of transformed fibroblasts appeared, in sharp contrast to the initial large cell size and flat “senescent” morphology (Fig. 5A, compare passage 10 to passage 0). Similar data were obtained in 3 independent Ras-expressing *FHL2^−/−^* cell lines (data not shown). When injected into nude mice, Ras-transduced *FHL2^−/−^* cells at passage 10 gave rise to tumors in 3/3 animals.

The transient cell cycle arrest of *FHL2^−/−^* cells contrasts with the permanent cell cycle arrest induced by Ras in primary rodent cells. To determine the role of p53 in the escape of Ras-transduced *FHL2^−/−^* cells from growth arrest, we measured the protein levels of p53 in *FHL2^−/−^*-transformed cells. After treatment with doxo, cell lysates were analyzed for p53 expression by immunoblotting. The basal levels of p53 in Ras-transduced *FHL2^−/−^* cells were higher than those in vector-transduced *FHL2^−/−^* cells, consistent with the induction of p53 in these cells by Ras (see Fig. 2D). However, p53 was still significantly induced by doxo in Ras-transduced *FHL2^−/−^* cells after reversal of the growth arrest, as well as in vector-transduced *FHL2^−/−^* control cell lines (Fig. 5B). These data suggested that p53 remained wt, which was further confirmed by sequencing p53 exons 4 to 10 in three different Ras-transduced *FHL2^−/−^* cell lines (data not shown). In contrast, no p53 induction by doxo was detected in Ras-transduced wt cells, as expected for the p53 mutant status (Fig. 5B). We then assessed the *INK4a/ARF* locus by examining expression of p16^INK4a^ and p19^ARF^ by immunoblotting. High levels of p16^INK4a^ were maintained in these cells after reversal, whereas a moderate increase of p19^ARF^ expression was detected in the same cell population (Fig. 5C). These data indicate that the reversal of Ras-imposed cell cycle arrest in *FHL2^−/−^* cells was not associated with disruption of the p19^ARF^/p53 pathway.

We have previously reported that FHL2 deficiency in immortalized MEFs is associated with low levels of cyclins D1, D2, D3 and E, and with hypophosphorylation of Rb [5]. The ability of Ras to activate cyclin D1 in both senescent and immortal cells [26], [34] prompted us to examine the expression of cyclin D1 and several other cell cycle regulators in Ras-tranduced *FHL2^−/−^* cells after reversal. We found that oncogenic Ras induced up-regulation of cyclin D1, but not D2 and D3 in wt cells (Fig. 5C), in agreement with previous report [43]. Strikingly, drastic induction was observed not only for cyclin D1, but also for cyclins D2, D3 and E in Ras-expressing *FHL2^−/−^* cells (Fig. 5C). In consequence, Rb phosphorylation was significantly augmented in these cells (Fig. 5C). Expression of H-RasV12 considerably potentiated the growth of *FHL2^−/−^* cells, although the proliferative rate remained slightly lower than that of Ras-transduced wt cells (Fig. 5D). Thus, in immortalized *FHL2^−/−^* cells, Ras-induced cell cycle arrest appeared to be a transient phenotype, and escape of the cell cycle arrest might be triggered by activation of G1-S cyclins by prolonged Ras expression, in turn leading to Rb phosphorylation. These observations indicate that if integrity of the p19^ARF^/p53 pathway is crucial for establishing cell cycle arrest in FHL2^−/−^ cell lines upon Ras expression, inactivation of Rb by increased cyclin/Cdk activity may be sufficient to reset their replicative lifespan. The resumed proliferative capacity diverts Ras activity to cell transformation.

### Ectopic expression of cyclin D1 restores the susceptibility of *FHL2^−/−^* cells to transformation by oncogenic Ras

**Figure 6:**
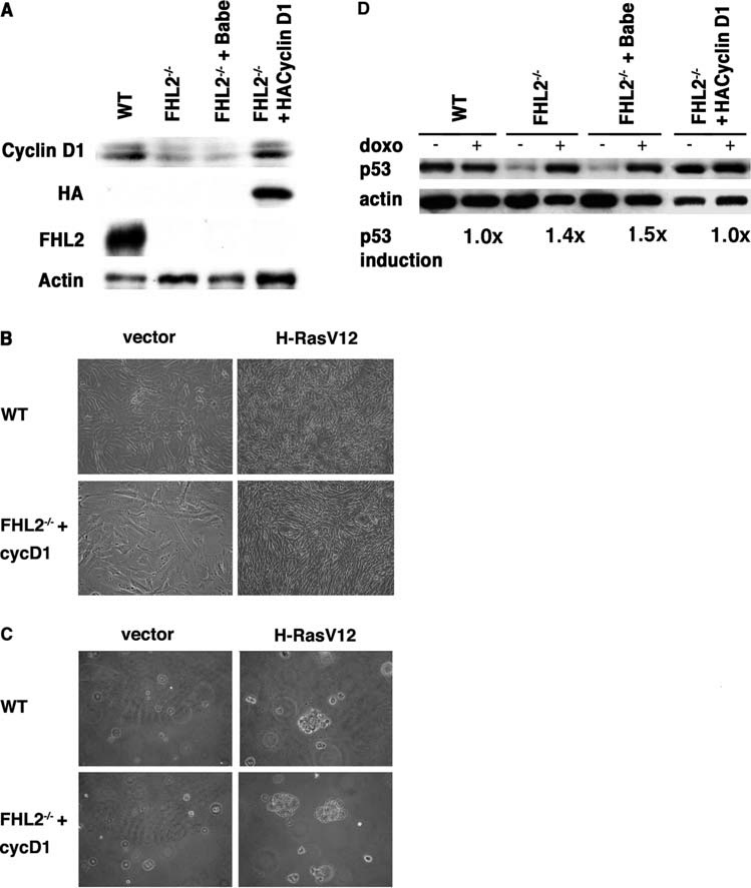
Immortalized *FHL2^−/−^* MEFs stably expressing cyclin D1 are susceptible to transformation by oncogenic Ras. (A) Increased cyclin D1 expression in immortalized *FHL2^−/−^* MEFs after introduction of cyclin D1-HA transgene was confirmed by immunoblotting with anti-cyclin D1 and anti-HA antibodies. (B) Representative phase contrast images of immortalized wt and *cyclin D1-HA/FHL2^−/−^* cell lines transduced with H-RasV12 or empty vector. (C) Soft agar assay. Cells were initially seeded at 5×10^4^ cells/plate and cultured for 10 days. Images are representative of three wt and two *cyclin D1-HA/FHL2^−/−^* cell lines transduced with H-RasV12. (D) Western blot analysis of p53 induction by doxorubicin in the indicated cell lines as described in Fig. 3A.

To directly evaluate the role of cyclin D1 in transformation of *FHL2*-null cells, we used immortalized *cyclin D1-HA/FHL2^−/−^* MEFs derived from primary *FHL2^−/−^* cells transduced with a retroviral vector for HA-tagged cyclin D1 (Fig. 6A) [5]. We have previously shown that expression of cyclins D1, D2, D3 and E and Rb phosphorylation as well a cell proliferation were restored to normal levels in these cells [5]. The effects of cyclin D1 restoration on the cellular response to mitogenic stimulation were evaluated after retroviral-mediated transfer of H-RasV12 in immortalized wt, *FHL2^−/−^*, pBabe-infected *FHL2^−/−^*and *cyclin D1-HA*/*FHL2^−/−^* cells. The Ras-transduced *cyclin D1-HA*/*FHL2^−/−^* cells showed a thin, elongated and spindle-like morphology similar to wt MEFs transformed by Ras (Fig. 6B), in contrast with the senescent morphology of Ras-transduced pBabe-infected *FHL2^−/−^* cells (see Fig. 2A and data not shown). When assayed for their ability to grow in soft agar, Ras-transduced *cyclin D1-HA*/*FHL2^−/−^* cells formed colonies at similar efficiency as wt MEFs (0.04%), whereas no colony was detected in *cyclin D1-HA*/*FHL2^−/−^* cells infected with the empty vector (Fig. 6C).

The observed susceptibity of *cyclin D1-HA*/*FHL2^−/−^* cell lines to Ras-induced transformation led us to examine the p53 status of these cells by comparing p53 expression levels in basal conditions and after doxo treatment. The p53 levels in *cyclin D1-HA*/*FHL2^−/−^* cells were as high as those in wt MEFs in the absence of treatment, and did not increase in response to doxo, reflecting mutant p53 (Fig. 6D), and similar results were obtained with two independent *cyclin D1-HA/FHL2^−/−^* cell pools (data not shown). In contrast, pBabe vector-infected *FHL2^−/−^* MEFs showed typical features of wt p53, with low basal levels of p53 and positive response to doxo stimulation. These findings suggest that restoration of cyclin D1 re-routed *FHL2^−/−^* cells to the common immortalization pathway associated with loss of p53 function and sensitivity to Ras transformation.

## Discussion

This study, showing that oncogenic Ras provokes a proliferative arrest in immortalized FHL2^−/−^ MEFs, uncovers a yet undocumented effect of Ras in the context of immortal mouse cell lines. We found that loss of FHL2 signalling confers immortalized MEFs the ability to retain an intact ARF/p53 checkpoint that is essential for Ras induction of cell cycle arrest, at difference with wt cell lines in which the ARF/p53 pathway is disrupted by mutation of p53 or deletion of the *INK4a* locus [29]. We provide evidence that the cyclin D/Rb pathway is critical in the regulation of cell cycle and transformation in *FHL2*-deficient cells.

### Low levels of cyclin D may override the requirement for disruption of the ARF/p53 checkpoint during establishment of FHL2^−/−^ cell lines

By genetic, biochemical and gene expression profile analyses, we show that immortalized *FHL2^−/−^* MEFs retain wt ARF and p53, implying alternative mechanisms for immortalization of *FHL2-*deficient cells. Although primary *FHL2^−/−^* MEFs undergo replicative senescence followed by emergence of immortalized variants with similar kinetics as in wt cells, immortalized *FHL2^−/−^* cell lines display unusual features. We first noticed dramatically reduced growth rate associated with down-regulation of cyclins D1, D2 and D3 (Fig. 5C and [5]). Spontaneous senescence occurs in the presence of mitogens [35]. Because D-type cyclins are critical cell cycle sensors of mitogens, decrease of all D-type cyclins may be sufficient for primary cells to bypass the growth arrest induced by the tissue culture environment. Such process might be selected in *FHL2*-deficient cells, with emergence of variants with low expression of cyclin D that may not fully sense hypermitogenic signals in the culture environment. By enabling cells to escape mitogen-induced senescence, this process might circumvent the need for loss of p53 or ARF. Thus in the absence of FHL2 signalling, we propose that decreasing sensitivity to mitogenic signals may, in part, account for an alternative mechanism of cell immortalization. This notion is supported by our observation that upon restoration of cyclin D1 expression in *FHL2^−/−^* cells, disabling the p53/ARF checkpoint becomes central to the establishment of *cyclin D1-HA*/*FHL2^−/−^* MEF lines as for wt cells. It suggests that the interplay between expression of mitogenic sensors and p53 functions might control cell growth or arrest in response to various stresses imposed by tissue culture. This hypothesis is consistent with previous observations showing that primary *cyclin D1*, *D2* and *D3* triple knock-out MEFs display reduced susceptibility to oncogenic agents [44].

### The integrity of the ARF/p53 pathway is responsible for Ras-induced cell cycle arrest in immortal FHL2^−/−^ cell lines

Immortalization is thought to endow cells with sensitivity to oncogene-induced transformation. Instead, the response of immortalized *FHL2^−/−^* MEFs to oncogenic Ras was characterized by cell cycle arrest associated with a senescent-like morphology and significant increase in p53, p21^CIP1^ and p16^INK4a^. Similar findings have been reported in normal human fibroblasts in which ectopic telomerase expression was sufficient for immortalization, but the introduction of Ras induced senescence [45]. In murine systems, it has been shown that cell immortality alone is not sufficient for transformation of *KSR1^−/−^* cells by Ras, and that disruption of the MAP kinase scaffold protein KSR1 directly blocks transduction of the Ras signal [46]. In *FHL2^−/−^* MEFs, resistance to Ras-mediated transformation might rather reflect the integrity of the ARF/p53 checkpoint, which senses inappropriate mitogenic signals and diverts cells to p53-dependent cell cycle arrest. This notion is supported by our finding that impairment of p53 function by overexpression of E6 or p53DD restores the susceptibility of *FHL2^−/−^* cells to Ras-induced transformation.

### Activation of cyclin D by Ras can override the p53-dependent growth arrest of FHL2^−/−^ cells

D-type cyclins represent the ultimate recipients of Ras oncogenic signal, and Ras can drive cell proliferation through all three D-cyclins [43]. Curiously, Ras activated all three D-cyclins in *FHL2^−/−^* cells, but failed to upregulate cyclins D2 and D3 in wt fibroblasts (this study and [43]), suggesting that FHL2 signalling has impact on all three D-cyclins. In turn, the dramatic increase in cyclin D led to efficient phosphorylation of Rb in *FHL2^−/−^* cells. The Rb tumor suppressor is required for senescence-associated heterochromatic foci (SAHF) formation and E2F target gene silencing, thus essential for the stability of senescent state in human fibroblasts [27]. However, SAHF formation in MEFs is less pronounced than in human fibroblasts [27] and senescent MEFs can rapidly re-enter cell cycle upon disruption of the p53 pathway [47]. These observations suggest that the control of the stability of senescence in mouse fibroblasts may be less dependent on Rb than in human fibroblasts and that p53 is required for both induction and maintenance of senescence in murine cells. It has been proposed that either inactivating p16^INK4a^, p21^CIP1^, p53 and Rb or re-activating cell cycle by c-myc, E2F, CDK2 and viral oncoproteins are required for overcoming senescence [48]. In the absence of p53 defects, the mechanisms involved in reversal of the cell cycle arrest in *FHL2*-deficient cells remain to be elucidated. A possible interpretation of our data might be that induction of G1-S cyclins by Ras eventually counteracts the p53-dependent proliferative block, leading to re-entry into the cell cycle and cell transformation.

FHL2 provides a unique mechanism in transcellular coordination and signal transduction by shuttling between focal adhesions and the nucleus. The up-regulation of FHL2 observed in human cancers suggests a potential role of FHL2 in the oncogenic process. This study demonstrates that the absence of FHL2 signalling provides a brake to cell transformation in tissue culture system. Suppression of FHL2 by antisense and siRNA methods has been shown to inhibit growth of gastric and colon cancer cell lines [22]. In mouse model, loss of FHL2 markedly decreases intestinal polyp formation induced by activation of the Wnt/β-catenin pathway (C.L., Y.N., F.L., S. Colnot, J. Chen, C. Perret, M.A.B. and Y.W., unpublished data). These results indicate that FHL2 signalling is important in the process of cell transformation and that its deficiency is benefitial for inhibition of this process. Treatment targeting FHL2 may thus provide a viable and specific strategy for cancer therapy.

## Materials and Methods

### Cell culture

Wild type and *FHL2^−/−^* MEFs have been described previously [5]. Cells were cultured in Dulbecco's modified Eagle's medium (DMEM) with 10% fetal bovine serum. Cell proliferation was determined by plating 2.5×10^4^ cells into 12-well plates in triplicate. Following initial plating, cells were harvested and counted for 6 days.

### Retroviral vectors and gene transfer

pWZLH-RasV12hygro was kindly provided by Dr. Scott Lowe. The pBabe-E6neo and pBabeH-RasV12puro retroviral vectors were gifts from Dr. Oliver Bischof. The pBabe-hygro p53DD construct from Dr. Bob Weinberg's lab [42] and the pBabe-puro-cyclinD1-HA from Dr. William Hahn's lab were obtained from Addgene (Addgene plasmids 9058 and 9050 respectively). The pBabe-FHL2puro was described previously [5]. Phoenix ecotropic virus packaging cells in 10 cm-culture dishes were transfected by phosphate calcium precipitation with 10 µg of retroviral vector. After 48 h, the virus-containing medium was filtered and supplemented with 8 µg/ml polybrene. For infection, the culture medium of target fibroblasts was replaced by virus-containing medium and incubated at 37°C for 3 h. The infection process was repeated three times, followed by drug selection.

### Transformation assays and tumorigenesis

For focus assays, cells were plated at 5×10^5^ cells in 10-cm culture dish. Cells were either transfected with 10 µg of either pCMV-H-RasV12 [49] or empty vector by using lipofectamine (Invitrogen), or infected with pBabe-E6neo and pBabe without selection. Cells were fed twice weekly and stained with Giemsa 3 weeks later. To generate stable cell lines expressing Ras and p53DD, MEFs were infected with virus-containing supernatant derived from Phoenix packaging cells transfected with the retroviral vectors pWZLH-RasV12Hygro, pBabeH-RasV12puro, and pBabe-hygro p53DD, followed by drug selection. For soft agar assays, cells were resuspended in 0.3% agarose in DMEM supplemented with 10% FBS at a density of 5×10^4^ cells per well in six-well plates and plated in triplicate onto solidified 0.6% agarose-containing medium. Colonies were counted and photographed 10 dyas after plating. For *in vivo* tumorigenesis assays, 5 to 10×10^6^ cells were implanted s.c. into the flanks of 8 week old female athymic BALB/c mice (Charles River Laboratories). Tumor growth was monitored for 2 to 3 weeks, and mice were sacrificed when the maximal allowable tumor size was achieved. No tumor developped during an observation period of 10 weeks in control groups. All animal procedures were carried out in accordance with French government regulations. Mice were housed under pathogen-free conditions.

### Immunoblotting

Cellular extracts were prepared in lysis buffer (50 mM Tris-HCl pH 7.5, 250 mM NaCl; 0.5% NP-40; 250 mM EDTA; 1 mM DTT; protease inhibitor cocktail (Roche)). Proteins were analyzed by immunoblotting as described previously [20]. The following primary antibodies were used: anti-H-Ras (sc-29), anti-p53 (sc-6243), anti-p21^CIP1^ (sc-397), anti-p16^INK4a^ (sc1207), anti-cyclin D1 (sc-450), cyclin D2 (sc-181) and cyclin D3 (sc-182) from Santa Cruz, anti-p19^ARF^ (ab80) from Abcam, anti-Rb (554136) from BD Pharmingen, anti-FHL2 (K0055-3) from MBL, anti-HA (Babco) and anti-actin (Sigma).

### p53 induction and immunoprecipitation

MEFs (1×10^6^ cells per well) were treated with 1 µg/ml doxorubicin and lysed 24 h later in IP buffer (50 mM Tris-HCl pH 7.5, 300 mM NaCl; 0.5% Triton ×100; 1 mM DTT; protease inhibitor cocktail (Roche)). The lysate was cleared by centrifugation and incubated with a mixture of monoclonal antibodies against p53 (0.5 µg; Pab 246 and Pab240, Santa Cruz) in the presence of protein A/G-Sepharose beads (Pierce). Proteins were analyzed by immunoblotting with anti-p53 antibody. Signals were quantified using Gene Snap/Gene Tools from SynGene.

### Direct PCR sequencing

DNA fragments spanning p53 exons 4–10 were amplified from 0.1–0.2 µg of genomic DNA with the following primers: for exons 4–5, 5′-TGGTGCTTGGACAATGTGTT-3′ (forward) and 5′-AGACGCACAAACCAAAACAA-3′ (reverse); for exon 6, 5′-GTAGGGAGCGACTTCACCTG-3′ (forward) and 5′-CAGAAGCTGGGGAAGAAACA-3′ (reverse); for exons 7–8, 5′-CCTTGTGCTGGTCCTTTTCT-3′ (forward) and 5′-ATGCGAGAGACAGAGGCAAT-3′ (reverse); for exon 9, 5′-TGTCCAGTGCTTCCATCTCA-3′ (forward) and 5′-GGAGGGAGGTCTGGGTAGAG-3′ (reverse); for exon 10, 5′-GGTGATGATGGTGATGGTGA-3′ (forward) and 5′-TATGGCGGGAAGTAGACTGG-3′ (reverse). For all exons, sequencing primers consisted of common sequences, are attached to the 5′ end of forward primers (5′-GTAAAACGACGGCCAGT-3′), and to the 5′ of the reverse primers (5′-CACACAGGAAACAGCTATGACCAT-3′). PCR products were purified by filtration on a MultiScreen®-HV plate (Millipore) loaded with Bio-Gel® P-100 Gel solution (Bio-Rad Laboratories). Nucleotide sequences were obtained from both ends using ABI PRISM BigDye Terminator sequencing kits and the 3130 Xl Genetic Analyzer (Applied Biosystems).

### Microarray analysis

Microarray analysis was performed as previously reported [5]. GSEA [37] was used to evaluate the correlation of a specific gene list with two different sample groups (genotypes). We used the signal2noise ratio as a statistic to compare specific and gene set permutations in order to evaluate statistical differences. Microarray data have been deposited in NCBI Gene Expression Omnibus (GEO Series accession number: GSE10902).

### mRNA analysis

Total RNA was isolated from wt and *FHL2^−/−^* MEFs with RNeasy minikit (Qiagen). 1 µg of RNA was reverse-transcribed using random primers and the Superscript Reverse Transcriptase (Invitrogen). The primer sequences used in real-time PCR are available upon request. The mean copy number of each gene from a triplicate determination was normalized to the mean copy number of 18S RNA.
